# Impact of the Guard Rings on Self-Induced Signal and Leakage Current in Trench-Isolated Low Gain Avalanche Diodes

**DOI:** 10.3390/s25103006

**Published:** 2025-05-10

**Authors:** Gordana Lastovicka-Medin, Gregor Kramberger, Jiri Kroll, Mateusz Rebarz

**Affiliations:** 1 Faculty of Natural Sciences and Mathematics, University of Montenegro, Dzordza Vashingtona, 81000 Podgorica, Montenegro; 2 Jozef Stefan Institute, Jamova Cesta 39, 1000 Ljubljana, Slovenia; gregor.kramberger@ijs.si; 3 Institute of Physics, Academy of Sciences of the Czech Republic, Na Slovance 2, 18221 Prague, Czech Republic; kroll@fzu.cz; 4 ELI Beamlines Facility, The Extreme Light Infrastructure ERIC, Za Radnicí 835, 25241 Dolní Břežany, Czech Republic; mateusz.rebarz@eli-beams.eu

**Keywords:** LGAD, inter-pixel region, trench isolation, self-induced signals, guard ring, bias ring, IV characteristics, TCT technique

## Abstract

In this contribution, we explored the interplay of guard ring (GR) configuration and isolation structures, as well as irradiation effects, which all together create a rich landscape of phenomena such as self-induced signals (“ghosts”) in trench-isolated Low-Gain Avalanche Diodes (TI-LGADs). The ghost effect is related to the increased surface current due to presence of SiO_2_ trenches (and defects) in studied diodes, but it is also affected by interplay between the guard ring(s) and the n^+^ bias ring, implanted in inter-pixel region of these devices. In double-trenched sensors, the n^+^ bias ring is inserted in between the two trenches. We present the investigation on the role of these structures on the self-induced signals in trench-isolated sensors from two different productions (RD50 and AIDAinnova). The sensors from the first production have multiple guard rings, whereas the second type of devices feature only one. Detailed examination of the ghost effect and leak current was performed when guard rings were left floating or connected to the pixels (brought to the same potential). The results show that guard ring configuration in trenched sensors can be critical for the leak current and the presence of a ghost signal. To our best knowledge, the latter problem has not been investigated yet.

## 1. Introduction

A Low-Gain Avalanche Detector (LGAD) is an n-on-p diode (n^++^/p^+^/p/p^++^ structure) with moderate internal gain enabled by a thin (~2 μm) highly doped p^+^ layer inserted beneath the n^++^ layer. These devices were designed as minimum ionizing particle time detectors (MIP TD) and high-granularity time detectors (HGDT) for Compact Muon Solenoid (CMS) and ATLAS experiments [1]. At the physical edge of the standard segmented LGAD, there are guard rings (GRs). These structures have the task of grading the voltage from the sensor edge to the first read-out pad, held at virtual ground by the read-out electronics. Each GR consists of an n^++^ doped implant, equipped with metal field plates. A p^+^ implant (either a p-stop or p-spray) is (in most designs) interposed between each pair of GRs, with the outer one left floating, and the inner one generally grounded in order to collect the leakage current generated outside the core region of the device. There are a few challenges in designing the periphery and interpad region in LGADs.

**Figure 1:**
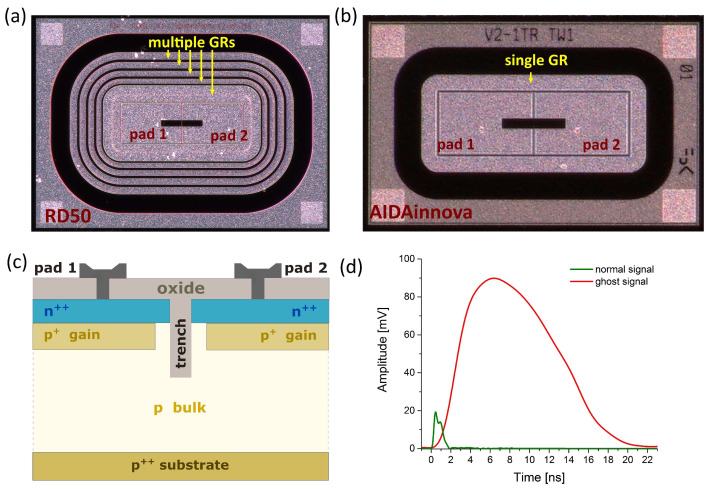
Layout of 1 × 2 pixels array of TI-LGADs: (**a**) RD50 sensor with multiple guard rings. (**b**) AIDAinnova sensor with single guard ring. (**c**) Cross-section of LGAD sensor with single trench. (**d**) Comparison of self-induced ghost signal in TI-LGAD device at 100 V with normal signal generated in pad of this sensor by 5 pJ laser pulse at the same bias.

1. Junction Termination Extension (JTE): In standard LGADs, the gain layer is surrounded by a deep n^++^ implant (JTE), which includes a metal field plate. The JTE is placed around each pad to prevent charge carriers generated in the interpad region from reaching the gain layer. When a particle traverses the gain layer, electrons immediately trigger the avalanche multiplication process. However, if charge carriers are generated in the interpad region, they must first drift to the gain implant, introducing a significant delay. The JTE mitigates this issue by confining the sensor’s active area to the regions where the gain implant is present.
2. P-stop isolation: In standard segmented LGADs, n^++^ implants must be electrically isolated from one another. This can be achieved using an additional p^++^ implant known as a p-stop. In some designs, isolation is enhanced by incorporating two p-stops along with a central n^+^ bias ring positioned between them. In order to improve the fill factor in segmented sensors, the isolation structures of small dimensions are desired. However, they lower sensor capability to hold high bias voltages. The p-stop structure is floating, so it floats to a potential between that of the n^++^ implant and the bias. There is, therefore, a strong electric field between the p-stop and the n^++^ pad: the shorter this distance, the higher the field. A few interesting features have been observed: (i) larger interpad distances lead to higher breakdown voltages, and (ii) the design with the widest interpad distance exhibits a breakdown due to gain. The high value of the p-stop doping is detrimental to sensor stability due to the presence of positive charges in SiO_2_ (at the Si-SiO_2_ interface). In contrast, low doping values increase the capability of holding high bias voltages even with short interpad distances. However, too low values of the p-stop doping might not assure pad isolation [2].
3. Guard rings: The role of GRs becomes increasingly complex as sensor thickness decreases. In ultrafast sensors, the lateral expansion of the depletion region is approximately equal to the sensor thickness. Consequently, floating GRs must be positioned within a lateral distance of about 2–3 times the active thickness from the innermost GR. Reducing the lateral spread of GRs increases the electric field between them, which can lead to premature breakdown. This poses a significant challenge in the design of ultrathin (~20–30 µm) ultrafast sensors. Even the innermost GR can be left floating, but in such a configuration, the adjacent pads will exhibit higher leakage current. The p-stops between GRs float to a potential between the bias voltage and ground; given the much-reduced thickness of the bulk, the p-stop in this case might float to potential quite close to the bias value. Under this condition, the sequence of GRs might not be able to sustain a large voltage drop. In RD50 TI-LGADs multiple GRs designs were applied (Figure 1a). In the standard version, the innermost ring is thicker than the others and can be contacted through an opening in the passivation. The rings should be isolated from each other; however, this depends on the depletion of the sensor.
4. GR optimization: The optimization of the sensor’s periphery is crucial to enable the sensor (if very thin (≤4 µm) or with a very narrow (few µm) interpad region) to operate efficiently up to very high fluence (~1 × 10^17^ 1 MeV n_eq_/cm^2^). If the peripheral region is too short, the depletion region can eventually reach the chip edge, leading to a high current injection into the core region. Devices with shorter trenches are even more vulnerable to this problem. When designing GR layout, while at the same time we are reaching sensor’s limits (geometric and operational) some sensor’s parameters may counteract and cancel each other. For instance, the optimization of the guard ring (GR) protection structure, especially when small substrate thicknesses are used, was extensively and systematically studied in a recent R&D batch produced at Fondazione Bruno Kessler (FBK) in the framework of the “eXFlu” project—INFN CSN5 grant for Young Researchers, where different optimization studies of GR structures for thin substrates (45, 30, 20 and 15 μm) up to high fluence (2.5 × 10^15^ 1 MeV n_eq_/cm^2^) were conducted [3,4]. Those studies have been enabled thanks to advanced Technology CAD (TCAD) modelling of different GR design strategies, accounting for the comprehensive bulk and surface radiation-induced damage effects. Typically, the sensor’s periphery region is (as already mention) composed by a collector ring, i.e., the bias ring, and a floating guard ring where GR ring is devised with both n-deep and p-stop implant. P-stop cuts off the possible buildup of the “inversion layer”, i.e., a surface leakage current path between the collector ring and the n-deep implant itself, thus avoiding connection between them. One of the most important massages and outcomes from the extensive study on GR structure layouts (on the samples from the eXFlu1 batch) was that after a fluence of 1 × 10^16^ n_eq_/cm^2^, a sensor periphery with floating GR seems to be more effective without any p-stop implant, benefiting from the concurrent action of bulk and surface damage to mitigate the build-up of the inversion layer. This result was very useful for the FBK decision on GR structure design implemented in the second TI-LGAD production (AIDAinnova batch). In the second TI-LGAD production (AIDAinnova batch), it was decided not to use the multi-GR- layout with multi-floating GRs anymore, but instead to use a single GR layout (shown in Figure 1b). The prevention of injection of surface leakage current in the core region of the sensor if the periphery region is too short [5] is still challenging task.
5. Trench Isolation: In 2019 the feasibility of implementing thin LGADs segmented using the SiO_2_ trench isolation technology was demonstrated and the first TI-LGAD batch was fabricated at Fondazione Bruno Kessler (FBK) within the RD50 Common project [6,7]. The wafer layout consists of pairs of pads (Figure 1a) with pad size 375 × 250 µm^2^, fabricated on 55 and 45 µm-thick p-epitaxial bulk. The layout splits implemented about 30 pairs, differing in the number of trenches (1 or 2), the dimension of the borders, trench process parameters, depth of trench (D1 < D2 < D3), and the distance between pixel borders (V1 < V2 < V3). Two different trench designs have been implemented in the first TI-LGADs production. The first one has a trench grid between pads (1TR), while in the second one, each pad is surrounded by an independent pair of trench rings (2TR). The nominal distance between the gain implants in the 1TR and 2TR designs is ~4 µm and ~6 µm [8], respectively. The second TI-LGAD production was released 2 years later, within the AIDAinnova project (Figure 1b) [9], also at FBK. In this production a medium depth of trench (equivalent of D2 in RD50 production) was chosen as standard while the width of trenches was varied. This production was also the first where carbon was implemented to increase the radiation hardness.

In our previous studies on TI-LGADs from both RD50 and AIDAinnova productions [10,11,12], we observed the extraordinary self-induced (without external stimulation) pulse signal, that we call “ghost”, appearing in all TI-LGADs after exceeding a certain value of applied bias. The example of a ghost signal with comparison to a standard signal generated in the same LGAD sensor is shown in Figure 1d. The properties of these signals, such as amplitude, duration, and occurrence rate, depend on many factors.

Trench configuration (1TR or 2TR), temperature, and irradiation affect the ghosts’ characteristics. The observed phenomenon is a complex interplay of many parameters where the manufacturing and process parameters for the studied sensors are not publicly accessible (due to non-disclosure agreements with vendor FBK). For this reason, performing simulations that would reliably support ghost effect elucidations is not yet possible. The current understanding of the discussed phenomena is fully based on the experimental observations, and a summary of the key results is presented in Section 3.1.1. Extending the previous research, here we investigate and discuss the influence of the GR configuration on the ghost signal in TI-LGADs. Sensors with multiple GRs (RD50) and a single GR (AIDAinnova) were investigated for this purpose. In addition, the impact of floating or biasing (connecting to pad) of the GRs on ghost effect is examined, and related changes in leakage current are discussed.

## 2. Materials and Methods

The sensors used in this study were designed and manufactured by Fondazione Bruno Kessler (FBK, Trento, Italy) within two different productions: RD50 and AIDAinnova. Every sensor had a form of 2 × 1 array (two pixels isolated by SiO_2_ trenches), as shown in Figure 1. Selected sensors were irradiated with neutrons at the Triga II Reactor of the Jozef Stefan Institute (JSI) in Ljubljana, Slovenia. A full list of the sensors examined in this study is presented in Table 1.

**Figure 2:**
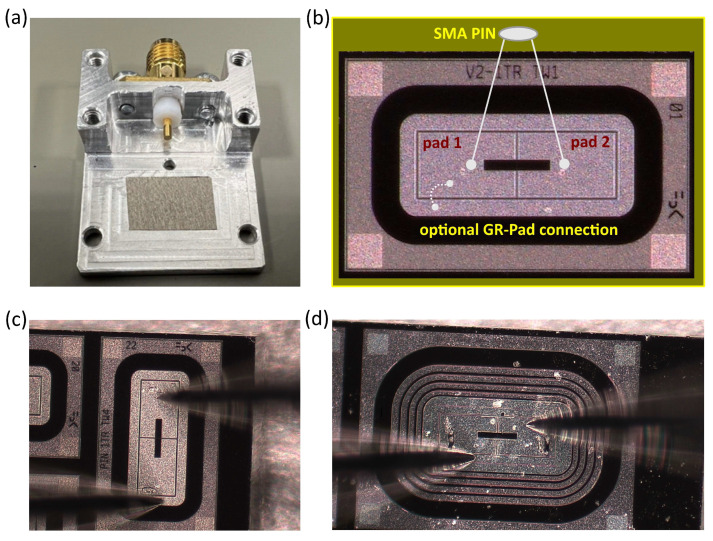
(**a**) Aluminum housing used for sensors mounting; (**b**) example 1 × 2 pixel sensor with indicated wire bonds; (**c**) AIDAinnova and (**d**) RD50 sensors during IV measurements.

The self-induced (ghost) signal generated upon the applied voltage was investigated by transient current technique (TCT) using the experimental setup at the ELI Beamlines facility (Dolni Brezany, Czech Republic) [13]. The aluminum housings, fabricated and designed at the Jozef Stefan Institute, were used for the samples mounting. The image of example housing is shown in Figure 2a. The samples were conductively connected to the housing itself (this all is then connected to the ground) and biasing of the sensors was achieved from the top side. Both signal pads of the sample were wire bonded to the pin of the SMA connector (which is electrically isolated from aluminum housing) as depicted in Figure 2b.

The pin was connected to HV power supply (EBS C0_30SHV, ISEG Spezialelektronik, Radberg, Germany) via a bias tee and the signal output was coupled directly to the oscilloscope (InfiniiVision DSOX6004A, 6 GHz, 20 GS, Keysight, Santa Rosa, CA, USA) without additional amplifiers. The generated waveforms were recorded at different bias up to maximal non-destructive values for a given sensor. The occurrence rate of the ghost signal was also measured by integrated counter with totalizer function (maximal measurable rate 25 MHz). The housings with sensors were placed on the cooling plate and all measurements were conducted at room (+20 °C) and low (−20 °C) temperature.

I–V measurements were performed in the Institute of Physics at Czech Academy of Science (Prague, Czech Republic) using an automatic probe station (TESLA200, FormFactor) with the triaxial chuck enclosed in the environmental chamber. The chuck with a golden surface (diameter of 200 mm) and implemented vacuum circuits was connected by the HV compatible triaxial cable with the HI of the source measure unit (SMU) via the 1 MΩ protection resistor. The testing pad and GR were contacted by the probes (see Figure 2c,d), which were entering the probe station environmental chamber through the so-called top hat. The probe contacting the testing pad was brought to the SMU LO by the triaxial cable. The GR of the sample was brought to the laboratory ground by the second probe, again by the triaxial cable. During the measurement, the relative humidity in the environmental chamber of the probe station was decreased to close to zero values.

All TCT and I–V measurements were performed for two different configurations of guard rings connections. In the first variant, all GRs in the sensors were left floating. In the second case, the innermost ring was brought to the pad potential by wire bonding of the corresponding areas (see Figure 2b).

## 3. Results

### 3.1. Ghosts in Trench-Isolated Sensors with Floating Guard Rings

#### 3.1.1. Previous Results

The occurrence of self-induced signals is a complex phenomenon involving various factors so we first provide a short summary of our previous results. We observed the first ghosts in the non-irradiated LGAD originated from RD50 production with double-trench IP isolation. In the next steps, we investigated the role of the following factors [10,11,12]:

- presence of the gain layer (LGAD vs. PIN)
- number of trenches (2TR vs. 1TR)
- irradiation effects (gain loss)
- temperature effects (room temperature vs. low temperature)
- gain layer carbonization (RD50 vs. AIDAinnova)

All these studies revealed that different types of ghosts exist in trench-isolated sensors depending on the sensor type and experimental conditions. Three main types of ghosts were identified.

- Type A: Signal appearing at low bias (typically 30–100 V in non-irradiated sensors) with occurrence rate decreasing with increasing bias (atypical features for auto-triggered signals in devices with internal gain and charge multiplication). This signal is strictly related to the presence of the gain layer. It is not observed in PINs (no gain) or irradiated LGADs that lost their gain (RD50 LGADs). We assumed that onset of this type of ghosts is around the GR structures. These discharges, experimentally verified and presented in [13], are enhanced by irradiation but significantly suppressed at low temperatures (tied to thermally activated processes, such as carrier generation via shallow traps or surface states).
- Type B: Signal appearing in medium bias range (typically >80 V in non-irradiated sensors) exclusively in 2TR LGADs. The signal of the same characteristics (amplitude and shape) can be also generated by laser stimulation when inter-pixel region is illuminated. This type of signal comes from the defects created between the trenches that can be thermally populated at room temperature (this signal is strongly suppressed at low temperature). Moderate dependence of its occurrence rate on bias is attributed to the presence of n^+^ implant placed between the trenches.
- Type C: Signal appearing at high bias (typically >200 V) exhibiting strong amplitude (order of magnitude higher than others). Occurrence rate of these events is strongly bias dependent and increases with rising voltage. This type of signal is associated with high-field phenomena such as impact ionization, avalanche processes, or enhanced trap-assisted carrier generation under strong electric fields. It is observed in all types of TI sensors when high enough bias value is reached.

The existence of individual types of ghost signals in different trench-isolated sensors is summarized in Table 2. In the following subsections, the new results for the sensors with different GRs configurations are presented.

#### 3.1.2. RD50 Sensors with Multiple GRs

**Figure 3:**
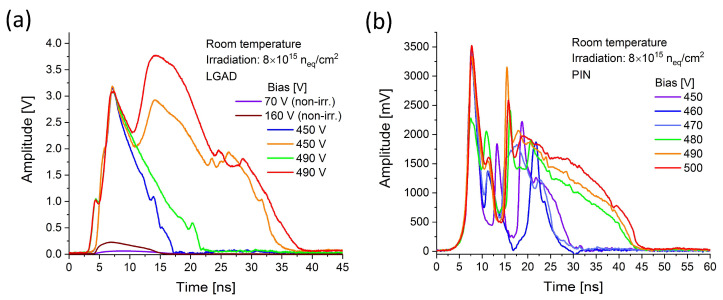
Ghost signals in irradiated and non-irradiated (**a**) LGADs (W11 C1-V2-2TR) and (**b**) PIN (W11 C1-V4-2TR) from RD50 production.

In case of LGADs from RD50 production, the ghosts appeared when the bias reached different level (depending on the sensor) in the range 30–90 V. These self-induced discharges are represented by the waveforms with width of several nanoseconds and amplitudes in the range of hundredths of mVs (see Figure 3a). Above the generation threshold, the ghosts are present in entire bias range, up to maximal non-destructive values (typically between 160 and 220 V, depending on the sensor and temperature). The ghost signal in this range vanishes completely in irradiated sensors. However, very strong self-induced discharges occur when the bias reaches the values close to the breakdown limits. In case of the sensors irradiated to fluence of 0.8 × 10^15^ n_eq_/cm^2^, where the breakdown bias is typically in the range 500–550 V, the ghosts appear at the voltage above 450 V. In comparison to their counterparts in non-irradiated samples, the representative waveforms have much higher amplitude (up to 4 V), multipeak shape and they are much broader extending up to 40–45 ns (see Figure 3a). The very similar signal was also observed in corresponding PIN irradiated at the same fluence (compare with Figure 3b). It is noteworthy that in non-irradiated PINs the ghosts were not observed even at the highest applicable bias and their presence in LGADs was attributed to the gain layer. However, in irradiated PIN, the self-induced signal is observed at the bias above 450 V in spite of lack of gain. Similarly to the irradiated LGADs, the ghosts appear at bias voltages very close to the onset of radiation-induced breakdown in the bulk. The multipeak character of this signal either in PIN or LGAD can be attributed to the presence of multiple GRs in all RD50 sensors (see Section 4 below).

#### 3.1.3. AIDAinnova Sensors with Single GR

**Figure 4:**
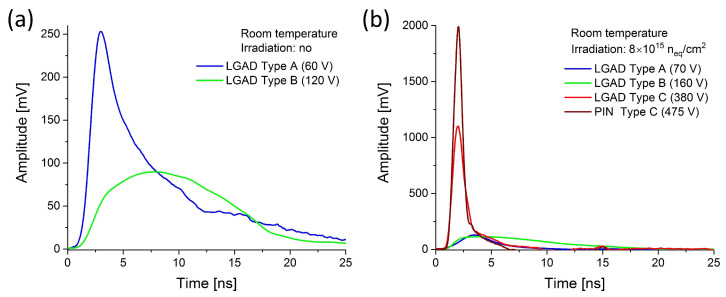
Ghost signals in irradiated and non-irradiated (**a**) LGADs (V2-2TR TW5 and V3-1TR TW5) and (**b**) PIN (1TR TW6) from AIDAinnova production.

In the trench-isolated sensors from the AIDAinnova production, two important aspects make them different from RD50 samples. LGADs from this production have a gain layer enriched with carbon. In addition, all AIDAinnova sensors feature only a single guard ring, contrary to the multiple GR structure in RD50 samples. These alterations can lead to different ghosts’ behavior. In non-irradiated TI-LGADs from AIDAinnova production, self-induced signal is quite similar to that observed in RD50. It appears above 30 V and increases with bias up to 90 V. Above that value, the signal changes its character, becoming broader and featuring lower amplitude. These signals, assigned before as Type A and Type B (see Section 3.1.1), are shown in Figure 4a. In the corresponding non-irradiated PIN sensor, the ghosts were not observed. After irradiation, the additional type of ghost, present at high bias values, appears in TI-LGAD but also in TI-PIN [11]. Although this signal exhibits very high amplitude (up to 2 V), it is very different from its counterpart recorded in RD50 irradiated sensors. The waveform is very narrow (see Figure 4b) and does not display the multipeak character. This feature is assigned to the fact that only a single guard ring is present in the sensors from AIDAinnova production. Although the difference between the two discussed productions lays not only in GRs configuration but also in gain carbonization (to reduce losses due to irradiation), the observed difference in ghost waveforms is manifested not only in LGADs but also in PINs without gain layer. Hence, the observed effect is only attributed to the difference in GRs.

### 3.2. Ghosts in Trench-Isolated Sensors with GR Connected to Pad

It was clearly observed experimentally that the self-induced signals in RD50 and AIDAinnova trench-isolated sensors behave differently. The observed differences indicate certain correlation with guard rings structure in both families of samples (multiple GRs in RD50 and single GR in AIDAinnova). Since, all observations were recorded for the sensors with floating rings, we decided to test what is the impact of GR potential for self-induced signals. For this purpose, the innermost GR in RD50 sensors and single GR in AIDAinnova samples were connected by additional wire with the neighbor pad (as presented in Figure 2b). After that, all the sensors were examined in exactly the same conditions (bias, temperature) as previously. It turned out that bringing the innermost GR to the same potential as the pad resulted in complete vanishing of ghosts. They were not observed in any circumstances in either LGADs or PINs.

### 3.3. I–V Characteristics for Trench-Isolated LGADs and PINs

Since the ghost signal vanishes in all trenched devices after GR connection, the only way to obtain some quantitative differences between investigated GR configurations is to monitor the related changes in leakage current.

#### 3.3.1. Non-Irradiated Trench-Isolated Sensors

**Figure 5:**
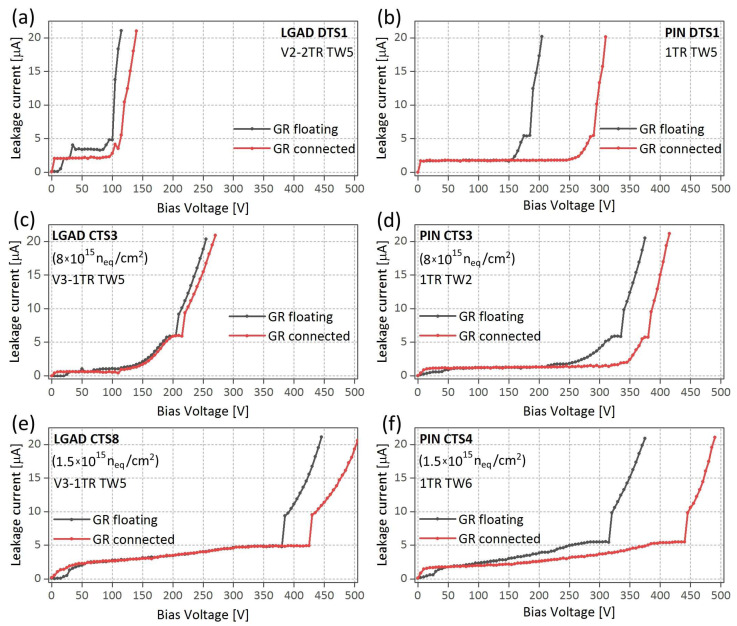
IV characteristics for non-irradiated (**a**,**b**), irradiated at 0.8 × 10^15^ n_eq_/cm^2^ (**c**,**d**), and irradiated at 1.5 × 10^15^ n_eq_/cm^2^ (**e**,**f**) trench-isolated sensors (LGADs and PINs) from AIDAinnova production.

IV characteristics of trench-isolated LGADs and PINs were first measured for non-irradiated detectors. In each case, IV curves were recorded for the sensor with floating GR and the same sample when GR is connected to its pad. For that purpose, two different sensors from AIDAinnova production were chosen, one LGAD (V2-1TR TW5 Cell-D TS1) and a corresponding PIN (1TR TW5 Cell-D TS1), both located on the same array. The results for these sensors are presented in Figure 5a,b. In the case of the LGAD, the breakdown voltage shifts about 20 V to higher values when the GR is at the same potential as the pad (Figure 5a). This effect is stronger in the case of the PIN, where the breakdown limit is shifted about 100 V (Figure 5b). All LGAD samples (including those not shown here) with floating GR exhibit a small local maximum in IV curve at the bias around 35 V. This effect disappears when the GR is connected to the pad and it is not observed in the PIN at all. It is probably related to the existence of different depletion regions in LGADs and their merging at certain bias (see discussion in Section 4.1.3).

#### 3.3.2. Irradiated Trench-Isolated Sensors

To examine irradiation impact on IV characteristics for the sensors with floating and pad connected GR, four sensors of different features were selected from AIDAinnova production. First, LGAD (V2-1TR TW5 Cell-C TS3) and corresponding PIN (1TR TW2 Cell-C TS3) irradiated to fluence of 0.8 × 10^15^ n_eq_/cm^2^ were tested. In both cases, the breakdown voltage was upshifted after connection of GR to pad (see Figure 5c,d). Stronger irradiation at fluency of 1.5 × 10^15^ n_eq_/cm^2^ does not change this general trend although it occurs with a different extent (see Figure 5e,f). In contrast to non-irradiated samples, the small peak around 35 V is not present in the curves for irradiated LGADs.

## 4. Discussion

### 4.1. Connection of Guard Ring to Pad

#### 4.1.1. Mitigation of Ghost Problem

It is known from the previous studies that application of floating GRs in the device periphery leads to the electrical field distortion in this region [5]. Previous experimental and theoretical works showed that increasing the reverse bias extend depleted region and above certain value can reach the n-type floating ring, which becomes biased by punch-through mechanism causing a redistribution of the electrostatic potential. When the guard ring is connected to the pad, it is at the same potential helping to control the electric field distribution. This leads to a more uniform electric field, reducing the likelihood of localized high-field regions that could trigger the ghosts. In our study, all the sensors with floating GRs exhibiting ghosts were reexamined in the same experimental conditions after connecting the innermost GR to the pad. This measure completely inhibited the ghosts’ occurrence in all studied sensors, even at the bias values close to the breakdown limits.

#### 4.1.2. Shifting the Breakdown Limits

Biasing of the GR affects clearly the leakage current and shifts the breakdown onset of the studied sensors. The IV curves for all the samples are shifted toward higher bias after connection of the GR with the pad (see Figure 5). The current measured in this configuration includes contributions from the leakage current across the detector volume, as well as the surface currents influenced by the GR. When left floating, the GR can accumulate the charge, creating localized field enhancements what can lower the maximal non-destructive voltage. The current measured in this case likely reflects the leakage current from the active region and the possible displacement currents induced by charge redistribution on the floating GR.

It is worth mentioning that, independently of GR configuration, the significant increase of the breakdown bias occurs after irradiation for all investigated sensors. Moreover, this shift is bigger for higher fluence. This effect is related to the acceptor removal mechanism which was observed in irradiated Si sensors and described previously [14]. In case of p-type Si, radiation exposure causes shallow acceptor dopants to lose their electrical activity. This deactivation, attributed to the formation of radiation-induced defects (e.g., boron-oxygen), leads to change in material conductivity and performance. Reduction in the effective doping concentration leads to a lower electric field, requiring a higher voltage to achieve the same level of charge multiplication, thus increasing the breakdown voltage.

#### 4.1.3. Smoothing the Local Anomalies

The local maxima in experimental IV curves of TI-LGADs with floating GR (Figure 5) are smoothed after ring-pad connecting. Their existence is a consequence of merging different depletion regions in the investigated samples which are very thin. Namely, in 45-micron sensors, thermal activation of the donor species can [15] result in the inversion of the initial p-type bulk (see Figure 1c) into the n-type material. Consequently, in the absence of an applied voltage, three depletion regions are present [16,17]:

- Top depletion region—located between the n^++^ top electrode and the p^+^ gain layer
- Middle depletion region—arising between the p^+^ gain layer and the n–converted substrate
- Bottom depletion region—formed between the n–converted substrate and the p^++^ backside electrode

Upon applying an external negative voltage (with the n^++^ top electrode at ground potential), all three depletion regions are expected to expand simultaneously, but at different rates. At the top, the depletion region between the gain layer and the top electrode grows both within the gain layer and toward the electrode. This expansion continues until the depletion covers the gap between the gain layer and the start of the implant for the top contact. In FBK sensors, this gap is approximately 2–3 µm and corresponds to the first inflection point in the IV curve, as the depletion dynamics change once expansion begins within the n^++^ top electrode. Simultaneously, a second depletion region forms at the bottom of the gain layer, expanding both toward the center of the gain layer and the backside. A critical event occurs when these two depletion regions meet within the gain layer, one expanding downward and the other upward. Together they continue to grow until they reach the third depletion region, which expands from the bottom of the sensor. Once full depletion is achieved, the sensor functions as a conventional LGAD. However, charge trapping during the depletion process can lead to transient effects, manifesting as spikes in the IV curve at the points where different depletion regions merge.

### 4.2. Floating Guard Rings Effects

Although biasing GRs by connection to the pad resolves some important issues, the design of the GR region is always a compromise between operation stability and ability to reach high efficiency across the detector. In standard LGADs, multiple floating rings are used to gradually reduce the electric field gradient toward the edges. This approach prevents field crowding, especially when the interpad distance is large (e.g., ~60 μm). However, in trench-isolated LGADs, floating rings can become less effective or even counterproductive because the trenches themselves help to confine the electric field.

#### 4.2.1. Impact on the Ghost Dynamics

In our previous study [12], three types of ghosts with different amplitude, shape, and occurrence rate dependence were observed, and they are briefly summarized in Section 3.1.1. Likely, all these types are affected by GR configuration, but the physical mechanisms governing these events are not yet fully understood. Nevertheless, the effects of multiple GRs on the dynamic of ghosts were mostly striking in irradiated RD50 TI-LGADs and TI-PINs where multipeak waveforms with extraordinary amplitude and duration were observed, as shown in Figure 3.

All RD50 samples have a multiple guard ring design (see Figure 1a). This means that in non-irradiated sensors, the floating GRs can act as charge collection points, affecting the electric field and signal properties. Their floating nature allows self-induced signals to occur due to charge redistribution. In irradiated samples, the radiation damage introduces trap states, which can enhance charge accumulation on the floating GRs. This can lead to (i) increased occurrence of self-induced signals, and (ii) enhanced amplitudes and prolonged signals (due to slow de-trapping from radiation-induced defects). Furthermore, the multiple peaks in waveforms (see Figure 3) likely arise from charge redistribution across multiple floating GRs, creating cascading signals.

AIDAinnova samples contain only a single GR (see Figure 1b). This solution may minimize electric field distortions, even under irradiation. This likely explains the lack of significant differences in self-induced signals between irradiated and non-irradiated TI-LGADs from this production (see Figure 4).

#### 4.2.2. Role of the Inner and Outer Rings

In case of multiple GRs, the ghosts’ mitigation requires biasing only the innermost ring. Therefore, we discuss here the role of inner and outer floating GRs on the ghosts triggering. At low bias, the inner guard rings (closer to active area) primarily manage the lateral electric field near the active area. They are more effective in preventing lateral leakage currents (e.g., surface leakage between the active area and the guard rings) because they are closer to the high-field region. However, their influence on bulk leakage currents is limited at low bias because the bulk is only partially depleted, and leakage currents are less pronounced in non-depleted regions. Surface defects (e.g., from irradiation) near the inner rings may still dominate leakage currents at low bias, as these regions may not yet be fully shielded. Contrary, the outer rings (closer to edge of device) play a secondary role because the depletion region does not extend to the edges. They mainly shield the edge from premature breakdown, but are less effective in mitigating leakage currents that originate near the active area. Their contribution to removing surface leakage is limited as the surface field near the edges is weaker at low bias. As bias increases and the bulk becomes fully depleted, the inner guard rings become less effective in controlling leakage currents because their primary role (managing the lateral electric field near the active area) diminishes when the depletion region expands. Bulk leakage currents dominate after irradiation, and the inner guard rings are less effective in mitigating these currents because they do not significantly impact the field in the bulk. Outer rings become more important at high bias as they handle edge effects and prevent field crowding near the periphery, which can lead to surface or lateral breakdown. They help to stabilize the electric field near the device edges, mitigating surface leakage currents caused by radiation-induced surface traps. Furthermore, their role in suppressing bulk leakage current is indirect, as they prevent edge-induced breakdown that could exacerbate overall leakage.

In irradiated devices, the depleted bulk contributes significantly to leakage current at high bias because of radiation-induced bulk defects (e.g., generation-recombination centers and trap-assisted tunneling). Guard rings have a limited direct impact on these currents, as they primarily affect lateral field distributions rather than the vertical field in the bulk. At high bias, the outer guard rings can reduce edge-related breakdown in the depleted bulk but cannot eliminate intrinsic leakage due to bulk defects. On the contrary, at low bias, leakage currents in the non-depleted bulk are lower because the generation of carriers is confined to a smaller volume. However, lateral leakage currents dominate in this regime, and the inner guard rings are more effective in mitigating these currents by controlling the lateral electric field near the active area.

## 5. Conclusions

Guard rings are critical in controlling the electric field distribution at the edges of the LGADs. When a GR is connected to the pad, it serves to stabilize the electric field and reduce edge breakdown by spreading the field more uniformly. This results in the following consequences:

- All ghosts appearing in all types of TI sensors vanish when GR is connected to the pad. This experimental fact indicates that surface current is large in the studied devices (and further also multiplied at the edge of the gain layer), causing the accumulation of charge in periphery region of pixels and around the GR, leading to the self-induced signals. Once the GR is connected, it drains out the surface current and prevents the appearance of the ghosts.
- Breakdown bias is higher when the GR is connected, which indicates the later onset of the charge multiplication in this case.

Leaving GRs floating in TI sensors is a source of different types of observed self-induced signals. Their exact character is related to several aspects but it is also dependent on the particular GRs configuration. This is especially the case of high-field ghosts (Type C) appearing at bias voltages very close to the onset of breakdown in the bulk (through the n^+^-p junction). Their signal is often identical to the induced current/signal from the ionizing particle. Their frequency and size increase with approaching the break down voltage. These events are therefore explained by thermally generated e-h pairs. The steep onset of ghost rate above certain bias voltage can be explained by the fact that even small inhomogeneity in doping of the gain layer leads to the point where the avalanche is triggered in certain area of the gain layer. A further increase of the bias voltage increases “the number” of those regions (rate increase) and also the size of signal. Here, the multiple GR configuration is reflected in a multipeak ghost signal, whereas a single GR results in a single peak waveform.

The influence of the GR structure on the ghosts appearing at lower bias is very complicated because of unpredictable biasing conditions of the GR and the pad (gain layer). It can lead to earlier achievement of the conditions required for ghost generation. If the GR and gain layer are at different potentials, there can be a large gradient between the two. In this case, depending on the geometrical properties (e.g., distance between the GR and the pad) the onset of ghosts can be reached earlier (at lower bias). The design of the interpad and the pad–GR region therefore plays a crucial role. While the shape of the signal in the case of the ghosts appearing at the pad is similar in shape of the particle/laser signal, the ghosts appearing in the GR–pad region may be different and very difficult to precisely explain, much more so if the GRs are left floating and the exact GR potential is difficult to know/calculate, particularly if the exact details of the region are not provided by the manufacturer.

Another effect that can be reduced is the interpad distance in TI-LGADs. In these sensors, the close proximity of active regions means the electric field profile between pads becomes more localized. This reduces the role of guard rings in spreading the field because the trenches themselves provide effective field isolation. With a smaller interpad distance, a single guard ring is sufficient to stabilize the field, as the reduced spacing minimizes the electric field difference across the region.

## Figures and Tables

**Table 1:** Trench-isolated sensors used in this study.

Production	Sensor	Wafer	Type	Irradiation
RD50	LGAD	W11	C1-V2-2TR	No
	PIN	W11	C1-V4-2TR	No
	LGAD	W3	C1-V2-2TR	0.8 × 10^15^ n_eq_/cm^2^
	PIN	W3	C1-V2-2TR	0.8 × 10^15^ n_eq_/cm^2^
AIDAinnova	LGAD	W1	V2-2TR TW5 Cell-D TS1	No
	PIN	W1	1TR TW5 Cell-D TS1	No
	LGAD	W1	V2-1TR TW5 Cell-C TS3	0.8 × 10^15^ n_eq_/cm^2^
	PIN	W1	1TR TW2 Cell-C TS3	0.8 × 10^15^ n_eq_/cm^2^
	LGAD	W1	V3-1TR TW5 Cell-C TS8	1.5 × 10^15^ n_eq_/cm^2^
	PIN	W1	1TR TW6 Cell-C TS4	1.5 × 10^15^ n_eq_/cm^2^

**Table 2:** Occurrence of different types of ghost signals in different trench-isolated sensors.

Sensor		RD50 Production (Multiple GRs, Non-Carbonized)		AIDAinnova Production (Single GR, Carbonized)	
		Non-Irradiated	Irradiated	Non-Irradiated	Irradiated
LGAD	1TR	Type C	Type A, C	no exp. data	Type A, C
	2TR	Type A, B, C	Type C	Type A, B, C	Type A, B, C
PIN	1TR	no ghosts	Type C	no ghosts	Type C
	2TR	no ghosts	Type C	no ghosts	no exp. data

## Data Availability

Data are available on the reasonable request.

## References

[1] Pellegrini G., Fernández-Martínez P., Baselga M., Fleta C., Flores D., Greco V., Hidalgo S., Mandić I., Kramberger G., Quirion D. (2014). Technology developments and first measurements of Low Gain Avalanche Detectors (LGAD) for high energy physics applications. Nucl. Instrum. Methods Phys. Res..

[2] Siviero F., Arcidiacono R., Borghi G., Boscardin M., Cartiglia N., Costa M., Dalla Betta G.F., Ferrero M., Ficorella F., Mandurrino M. (2024). Design optimization of the UFSD inter-pad region. Nucl. Instrum. Methods Phys. Res..

[3] Croci T., Morozzi A., Sola V., Asenov P., Fondacci A., Giordanengo S., Borghi G., Vignali M.C., Paternoster G., Boscardin M. (2023). TCAD optimization of LGAD sensors for extremely high fluence applications. J. Instrum..

[4] Croci T., Morozzi A., Fondacci A., Lanteri L., Siviero F., Sola V., Ferrero M., Menzio L., Mulargia R., Arcidiacono R. (2024). Advances in the TCAD modelling of non-irradiated and irradiated Low-Gain Avalanche Diode sensors. J. Instrum..

[5] Fernández-Martínez P., Flores D., Hidalgo S., Greco V., Merlos A., Pellegrini G., Quirion D. (2016). Design and fabrication of an optimum peripheral region for low gain avalanche detectors. Nucl. Instrum. Methods Phys. Res..

[6] Paternoster G., Borghi G., Arcidiacono R., Boscardin M., Cartiglia N., Centis Vignali M., Dalla Betta G.F., Ferrero M., Ficorella F., Mandurrino M. (2021). Novel strategies for fine-segmented Low Gain Avalanche Diodes. Nucl. Instrum. Methods Phys. Res..

[7] Paternoster G., Borghi G., Boscardin M., Cartiglia N., Ferrero M., Ficorella F., Siviero F., Gola A., Bellutti P. (2020). Trench-Isolated Low Gain Avalanche Diodes (TI-LGADs). IEEE Electron. Device Lett..

[8] Senger M., Macchiolo A., Kilminster B., Paternoster G., Centis Vignali M., Borghi G. (2023). A comprehensive characterization of the TI-LGAD technology. Sensors.

[9] Consortium for Advancement and Innovation for Detectors at Accelerators (AIDAinnova), AIDAinnova Website. https://aidainnova.web.cern.ch.

[10] Lastovicka-Medin G., Kramberger G., Mrkic D., Baletic V., Backovic V., Kroll J., Andreasson J., Rebarz M. (2025). Dynamic quenching of self-induced and self-sustaining avalanches in double-trenched LGADs. J. Instrum..

[11] Laštovička-Medin G., Manojlović M., Backović V., Mrkić D., Baletić V., Kramberger G., Kroll J., Laštovička T., Andreasson J., Rebarz M. (2025). A ping-pong self-sustaining avalanche in TI-LGAD sensors. Eur. Phys. J. Spec. Top..

[12] Lastovicka-Medin G., Mrkic D., Baletic V., Kramberger G., Kroll J., Rebarz M. Ghosty TI-LGAD, 2nd DRD3 Week, CERN, 2–6 December 2024. https://indico.cern.ch/event/1439336/contributions/6242213/attachments/2977669/5242214/CERN_2ndWeek_DRD3_Ghosts_Irradiated_Ti-LGADs.pdf.

[13] Laštovička-Medin G., Rebarz M., Kramberger G., Kroll J., Kropielnicki K., Laštovička T., Precek M., Andreasson J. (2022). Femtosecond laser studies of the Single Event Effects in Low Gain Avalanche Detectors and PINs at ELI Beamlines. Nucl. Instrum. Meth. A.

[14] Moll M. (2020). Acceptor removal—Displacement damage effects involving the shallow acceptor doping of p-type silicon devices. Proceedings of the 28th International Workshop on Vertex Detectors, Lopud, Croatia, 13–18 October 2019.

[15] Centis Vignali M. (2025). Personal Communication.

[16] Bharthuar S., Ott J., Brücken E., Gädda A., Kirschenmann S., Golovleva M., Luukka P.R. Effect of Thermal Donors Induced in Bulk and Variation in P-stop Dose on the No-gain Region Width Measurements of LGADs. Proceedings of the 29th International Workshop on Vertex Detectors (VERTEX2020).

[17] Gkougkousis V. (2025). Personal Communication.

